# Mutations in ribosomal protein uS5 alter translation fidelity and mutagenesis in *Pseudomonas putida*

**DOI:** 10.1128/jb.00334-25

**Published:** 2025-11-12

**Authors:** Karl Jürgenstein, Heili Ilves, Carol Luhaäär, Age Brauer, Jaanus Remme, Maia Kivisaar

**Affiliations:** 1 Department of Genetics, Institute of Molecular and Cell Biology, University of Tartu 37546, Tartu, Estonia; 2 Department of Bioinformatics, Institute of Molecular and Cell Biology, University of Tartu 117223, Tartu, Estonia; 3 Department of Molecular Biology, Institute of Molecular and Cell Biology, University of Tartu 117223, Tartu, Estonia; National Institutes of Health, Bethesda, Maryland, USA

**Keywords:** translation accuracy, spectinomycin resistance, dual-luciferase assay, mutation rate, evolvability of bacterial populations

## Abstract

Errors in protein synthesis can influence cellular fitness and, in some cases, affect genome stability. While this connection has been explored, most investigations have focused on Enterobacteriaceae such as *Escherichia coli*, leaving open the question of how translational fidelity shapes mutagenesis in other bacterial groups. Would a metabolically versatile, stress-tolerant soil bacterium behave differently? We focused on the small-subunit ribosomal protein uS5 (*rpsE*) because, despite its role in decoding accuracy, its potential as a mutator locus has not been evaluated. To explore this, we isolated several mutants of ribosomal protein uS5 (*rpsE*) in *Pseudomonas putida*, all carrying amino acid substitutions or deletions in loop 2, via spectinomycin selection, and then quantified their effects on frameshifting and stop-codon readthrough using dual-luciferase reporters. In parallel, we measured the rate and spectrum of spontaneous rifampicin resistance (*rpoB*) mutations. The fitness costs of these uS5 alleles were also assessed through growth and stress tolerance assays. Several mutants displayed divergent decoding phenotypes, including both error-prone and error-restrictive profiles, and the increase in translational errors was often, but not universally, coupled with elevated mutation rate. This approach probes which uS5 perturbations confer a *bona fide* mutator phenotype and how decoding errors bias mutation outcomes. By extending fidelity mutagenesis studies beyond enterobacteria, our work identifies uS5 as a previously unrecognized mutator locus in *P. putida* and illuminates the nuanced coupling of translation accuracy and evolvability in a key environmental microbe.

**IMPORTANCE:**

The accuracy of protein synthesis is essential for cellular integrity, yet its influence on genomic stability remains poorly understood, especially outside model organisms. By examining mutations in ribosomal protein uS5 of *Pseudomonas putida*, a bacterium adapted to environmental stress, we reveal how altered translation fidelity can modulate spontaneous mutagenesis. Some mutations increased translational errors and mutagenesis, while others decoupled these phenotypes, highlighting mechanistic complexity. These findings suggest that the link between translation fidelity and evolvability may be context-dependent, shaped by both ribosomal structure and environmental adaptation. Our work expands fidelity mutagenesis studies into non-enteric bacteria and offers insights into how translation errors may contribute to adaptive potential in fluctuating environments.

## INTRODUCTION

Accurate protein synthesis is vital for maintaining the adequate function of the proteome—not only to preserve its critical functions but also to safeguard the cellular homeostasis by preventing the accumulation of errors. Aberrant proteins may disrupt essential pathways, inhibit stress responses, and burden the energetic balance of cells, leading to decreased fitness. Erroneously translated proteins can misfold, aggregate, engage in wrong interactions, and saturate protein quality control machinery, resulting in proteotoxic stress. Moreover, since DNA replication is inherently dependent on the precise function of proteins, a compromised proteome may influence genome stability (1). On the flip side, this may, in turn, provide selective advantages for an organism under specific conditions to rapidly adapt to the environment. Some errors might be advantageous by initiating stress-induced mutagenesis, thereby increasing the likelihood of adaptation of bacteria by natural selection (2–4).

While protein synthesis and DNA replication can be viewed as distinct processes, evidence has emerged of a correlation between errors of these reactions (5, 6). Broadly, mutations arise through diverse mechanisms that perturb the information stored within DNA. Spontaneous or induced in their origin, these alterations form the substrate for genetic variability. In bacteria, DNA replication yields errors at a rate of about 10^–9^–10^–11^ per nucleotide per generation (7, 8), while errors during translation are several orders of magnitude more common, with ribosome slipping or incorporating an incorrect amino acid at a frequency of 10^–3^–10^–4^ per codon (9, 10), and stop-codon readthrough (nonsense suppression) is on the order of 10^–3^–10^–2^ per decoding event (11, 12). From an evolutionary standpoint, this makes sense, since translation errors tend to be transient, affecting only the function (and the consequences) of the incorrectly synthesized protein. Mutations, on the other hand, become fixed in the genome and, depending on their effect on the cell (deleterious, neutral, or beneficial), affect all subsequent generations. From an evolutionary perspective, organisms may benefit from either a “soft” version of adaptation, where the changes in the proteome create a phenotypic adaptation without a fixed presence in the genome, perhaps better suited for a fluctuating environment, or a “hard,” genetically fixed version of adaptation (13).

*Pseudomonas putida* is a soil bacterium renowned for its metabolic versatility and stress tolerance that thrives in fluctuating environments and has been studied extensively with respect to biodegradation of pollutants and the stress tolerance mechanisms (14–16). Its translational fidelity profile also markedly differs from that of *Escherichia coli* (17). An increase in mutations and translational errors has been shown in *P. putida* strains with tRNA pseudouridylation defects, further illustrating the potential for translational fidelity to influence mutagenesis beyond its immediate role in protein synthesis (17, 18).

Ribosomal proteins in the small subunit help guard against translational errors by stabilizing rRNA and modulating tRNA-mRNA interactions (19). Among these is ribosomal protein uS5, which together with proteins uS4 and uS12, is critical in reading frame maintenance: the uS4-uS5 interface regulates the transition between the ribosome’s “open” and “closed” conformations during tRNA selection (20, 21). This conformational change is essential for accurate decoding as it allows the ribosome to distinguish between cognate and non-cognate tRNAs. Mutations in *rpsD* (encoding ribosomal protein uS4) that create ribosomal ambiguity (Ram) mutants, characterized by increased translational errors, also elevated spontaneous mutation rates (22). When uS5 is mutated, it has been shown to evoke ribosome assembly defects, cold sensitivity, spectinomycin resistance, and Ram phenotypes in *E. coli*. While most uS5 mutations decrease translational accuracy, some error-restrictive mutations have also been described, highlighting the complex role of uS5 in maintenance of translational fidelity (23, 24). So far, no secondary functions of the uS5 protein, where it might interact with DNA directly, have been reported. As such, uS5 protein serves as a prime candidate to test whether translational errors are contained in the moment and only affect current cellular functions, or have the ability to influence evolutionary trajectories.

In this study, we investigate whether compromised translational fidelity confers a *bona fide* increase in mutation rate in *P. putida*. To this end, we generated a panel of ribosomal protein uS5 mutants by spectinomycin selection, as resistance to this antibiotic is associated with altered decoding accuracy. Translational frameshifting and stop-codon readthrough were quantified using dual-luciferase reporters, while spontaneous rifampicin resistance mutant frequency and associated *rpoB* mutation spectrum were determined. In addition, the impact of uS5 mutations on bacterial growth characteristics was assessed.

## RESULTS

### Generation of uS5 mutants by spectinomycin screen

**Fig 1:**
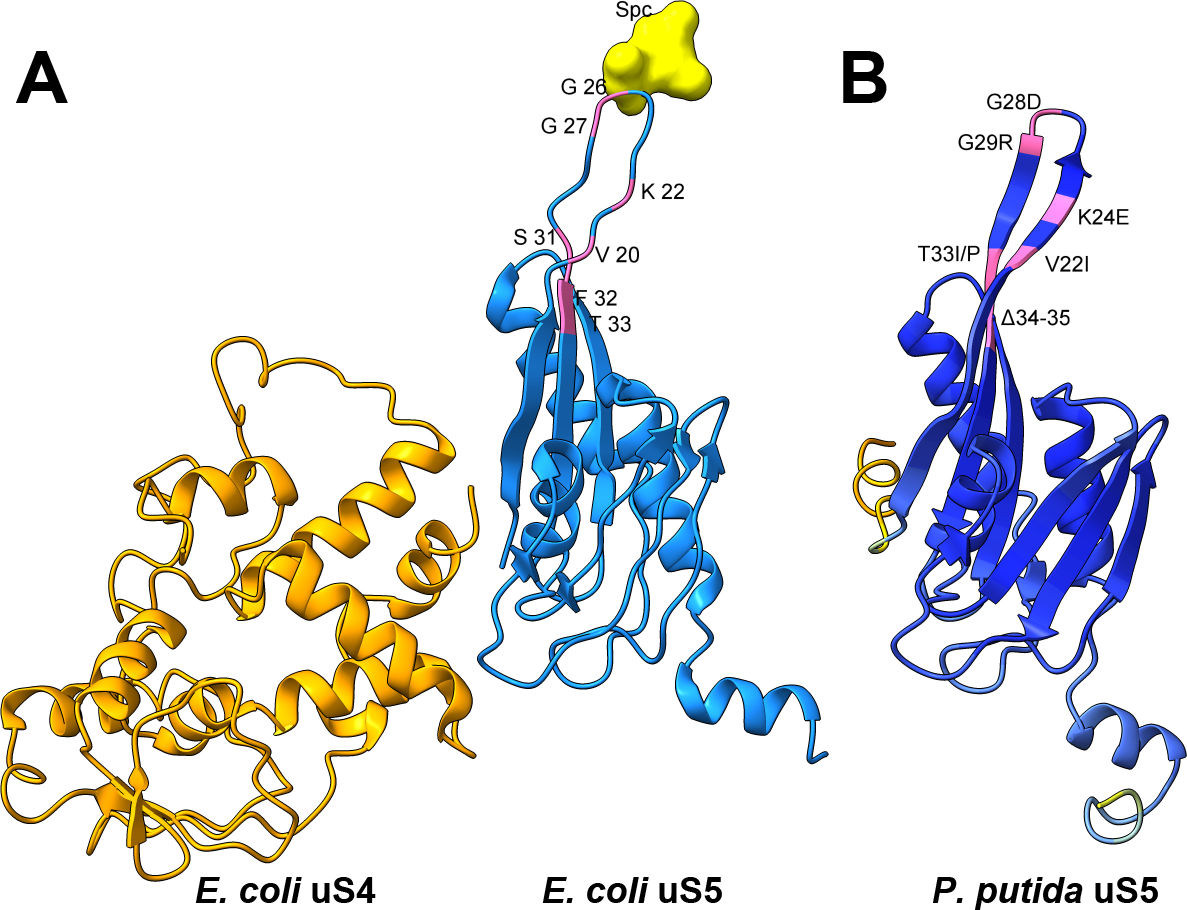
Structural context of uS5 mutations and spectinomycin binding. (**A**) *E. coli* uS4 (orange), uS5 (light blue), and spectinomycin (yellow) positioning within 30S ribosome (PDB 4V56). Residues in uS5 corresponding to the *P. putida* variants are highlighted in pink. (**B**) AlphaFold prediction of *P. putida* uS5 (UniProt Q88QL8; AF model) colored by predicted local distance difference test (pLDDT; dark blue = very high confidence; yellow = low confidence; orange = very low confidence), with mutated residues highlighted in pink. The figures were produced with UCSF ChimeraX (27).

To isolate uS5 mutants, a selection strategy based on spectinomycin resistance was employed, following a modified version of the method described by Kamath et al. (25). *P. putida* PaW85 wild-type cells were grown overnight at 30°C, and 100 µL aliquots were plated onto LB-spectinomycin (1,750 μg/mL) selective plates. Spontaneously arising mutants that emerged on selective plates during 48 h of incubation were subjected to sequencing of the *rpsE* gene to confirm the presence of mutations. In total, 7 uS5 mutants with different mutations in the *rpsE* gene were isolated, and the lack of additional mutations in the genome was confirmed by whole-genome sequencing (WGS; reads deposited under BioProject accession PRJNA1337407). Mutant strains were named after the amino acid change (V22I, K24E, G28D, G29R, T33I, T33P, and ∆34-35). Mutated residues were mapped onto the AlphaFold-predicted structure (26) for visualization and, by sequence alignment, onto the *E. coli* uS5 structure within 30S ribosome (PDB 4V56) to provide structural context alongside uS4 and spectinomycin (Fig. 1).

### Translational accuracy in *P. putida* is affected by amino acid alterations in the uS5 protein

**Fig 2:**
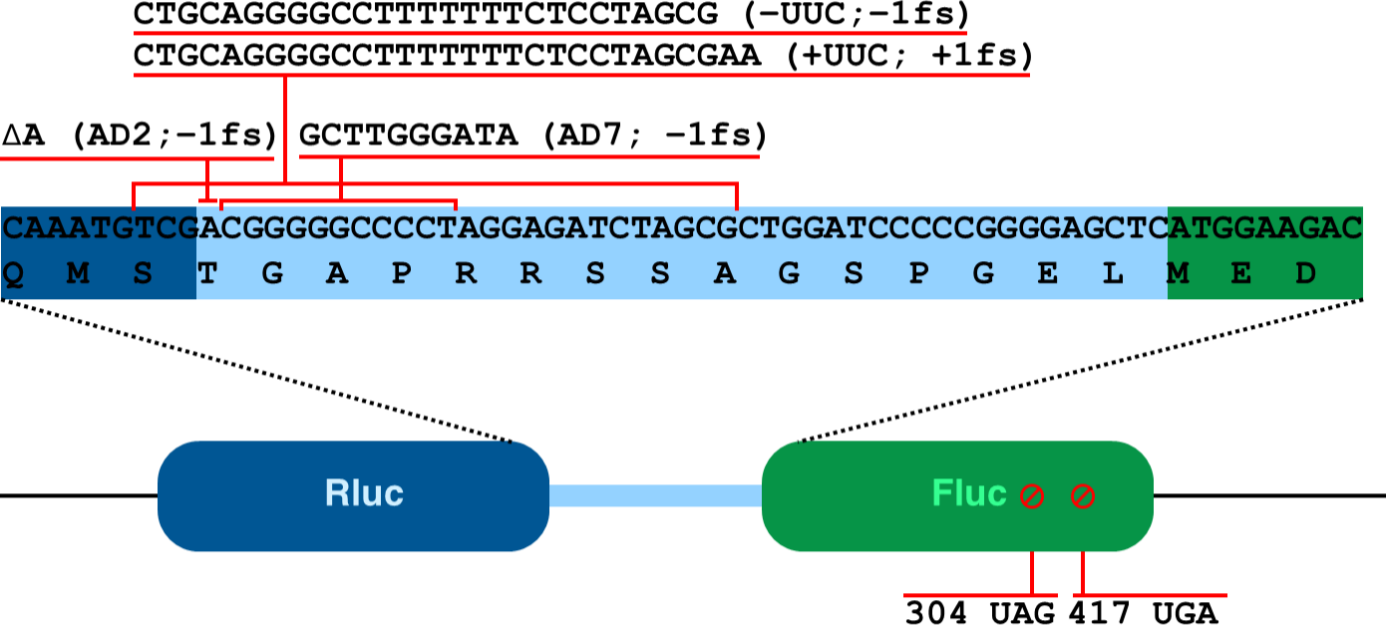
Schematic representation of the dual-luciferase assay system reporter used to measure the translational fidelity. Rluc (dark blue) and Fluc (green) form a fusion protein connected by a short linker region (light blue). Nucleotide and amino acid sequence of the linker region is shown with adjacent regions of Rluc and Fluc highlighted. Insertions and deletions introduced into the system to measure translational frameshifting are shown with red lines above the sequence, and premature stop codons with their positions are shown below.

**Fig 3:**
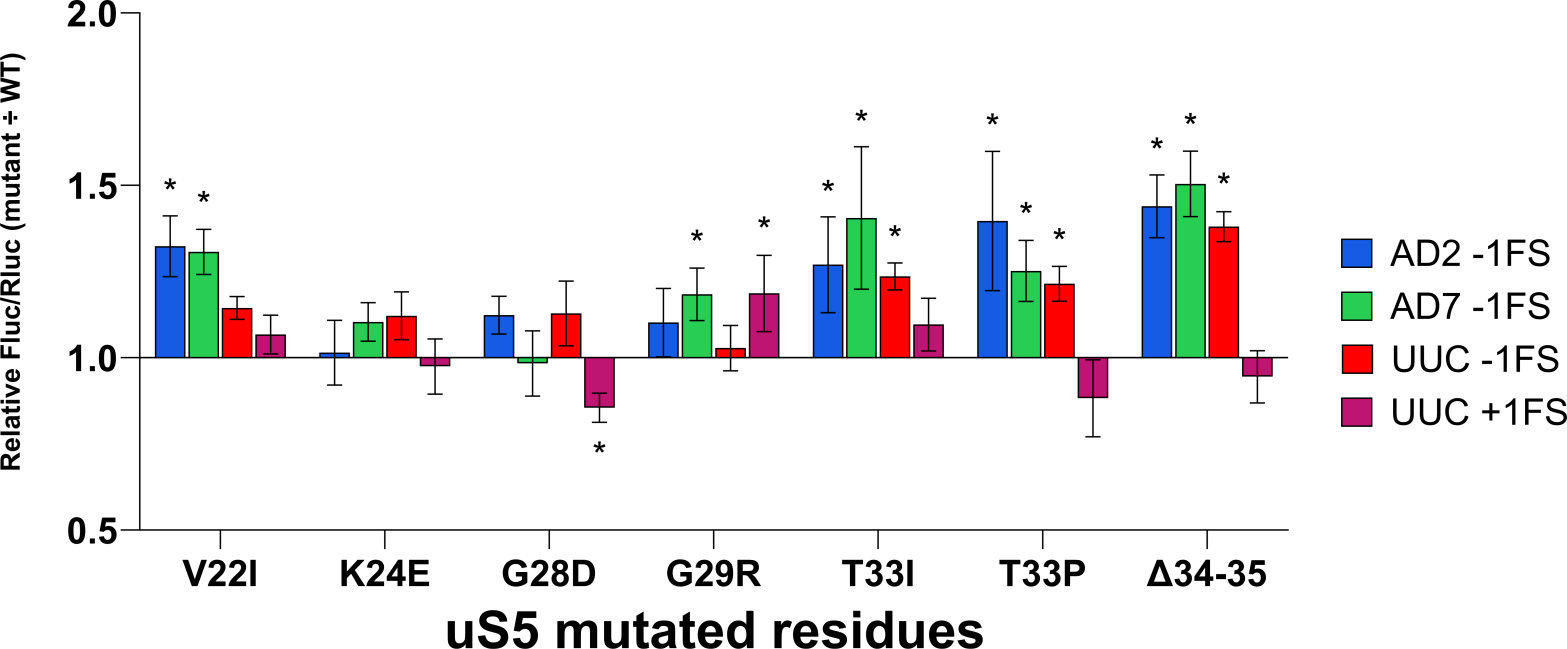
Effect of altered ribosomal protein uS5 on translational frameshift frequency relative to wild type in *P. putida* PaW85. Strains carrying uS5 mutant alleles have been normalized against the wild-type strain within each reporter (depicted in different colors). Error bars represent CI 95%, * indicates *P*-value < 0.05 compared to the respective wild type, *n* ≥ 11.

**Fig 4:**
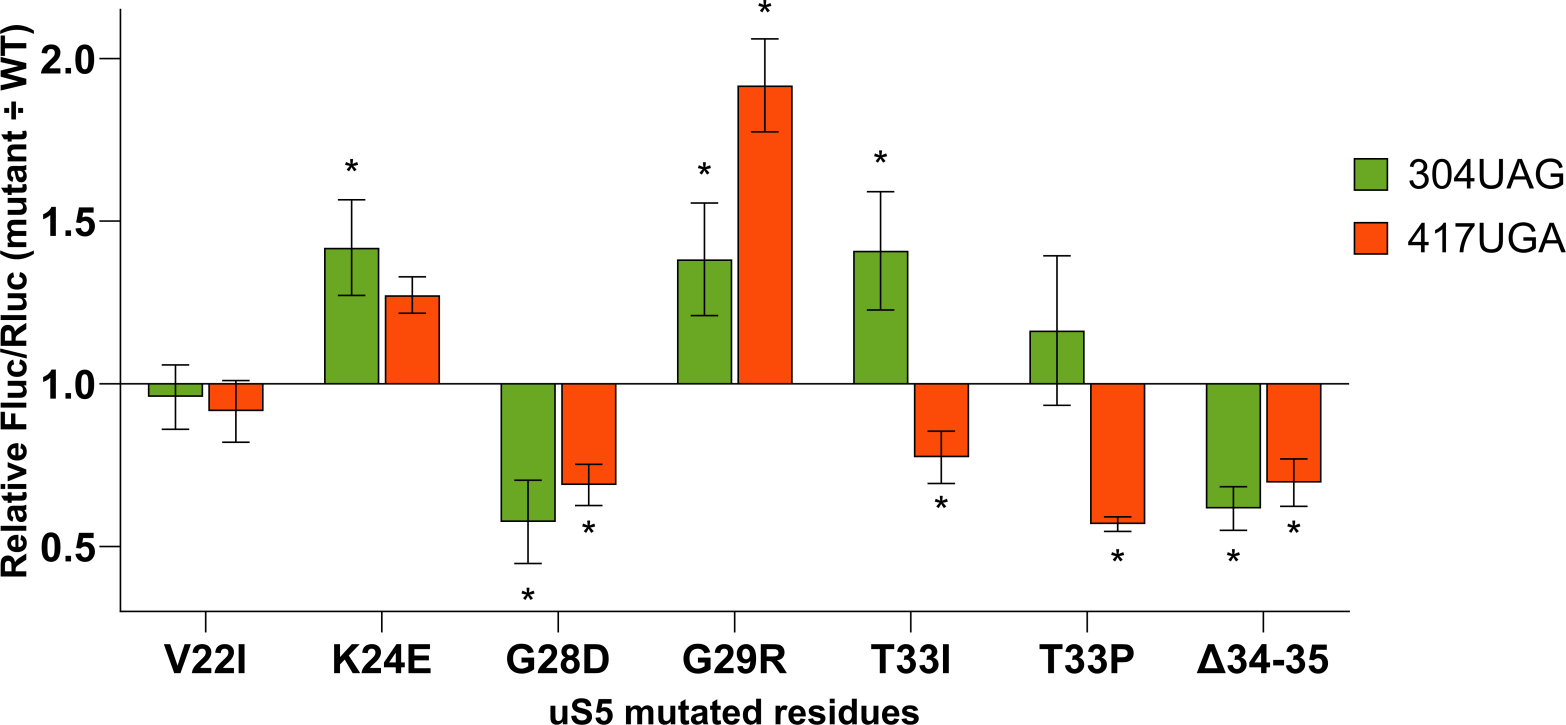
Effect of altered ribosomal protein uS5 on translational stop-codon readthrough frequency relative to wild type in *P. putida* PaW85. Strains with uS5 mutant alleles have been normalized against the wild-type strain within each reporter (depicted in different colors). Error bars represent CI 95%, ‘*’ indicates *P*-value < 0.05 compared to the respective wild type, *n* ≥ 11.

Mutations in ribosomal proteins uS4, uS5, and uS12 change translation accuracy in *E. coli* (19). Mutations in uS4 (*rpsD*) and uS5 (*rpsE*) usually cause an increased frequency of missense and frameshift errors (20). We have analyzed translation fidelity in *P. putida* strains with *rpsE* gene (uS5) variants exhibiting spectinomycin resistance. Translational accuracy was assessed by employing a dual-luciferase assay system (Fig. 2). Six different reporters were used, four to assess the frequency of frameshift at known slippery sites (one + 1 fs and three −1 fs) and two to measure the frequency of stop-codon (UAG and UGA) readthrough. Seven strains with altered uS5 proteins and the wild-type *P. putida* PaW85 strain were analyzed. Mutant strain Fluc/Rluc ratios of each reporter variant were normalized to the respective (same reporter variant) mean Fluc/Rluc ratio of the wild-type strain. Higher values indicate greater error in the corresponding assay (+1/−1 frameshifting or UAG/UGA readthrough). Results are shown in Fig. 3 (frameshift reporters) and Fig. 4 (stop-codon readthrough reporters).

Regarding translational frameshifting, mutated uS5 proteins tended to elevate the error rate compared to wild type, with only a statistically significant decrease in error rate (+1 fs in the G28D strain). Alterations around positions 33–35 seemed to affect the error rate more substantially than alterations closer to the N-terminus of uS5; however, only −1 frameshifting was significantly affected in these mutants. The highest cumulative effect was measured in the ∆34-35 strain, in which all the measured −1 frameshifts had increased by 1.3–1.5× compared to the wild type. It is worth noting that +1 frameshifting was significantly affected only by altered glycine residues in positions 28 and 29, but interestingly, not in a unidirectional manner: an increase in error rate in the case of G29R and a decrease in G28D was observed.

The effect of mutations in the uS5 protein on stop-codon readthrough was more varied. Similar to the +1 frameshifting, both UAG and UGA readthrough were increased in the G29R strain, with UGA stop-codon misread at about 1.9× of wild-type frequency. At the same time, the G28D substitution on the neighboring residue caused a significant decrease in translational readthrough. Deleting the residues 34–35 also resulted in a similar reduction of readthrough on both UAG and UGA stop codons. Next to it, substitutions of the threonine at the 33rd position by either isoleucine or proline resulted in decreased UGA readthrough and increased UAG readthrough (albeit the increase was statistically nonsignificant when replaced by proline). While frameshifting was unaffected by the K24E change, UAG readthrough increased to about 1.4× of wild type. Contrarily, the V22I alteration, which increased −1 frameshifting on AD2 and AD7 sequences, did not affect stop-codon readthrough.

Taken together, mutations in the uS5 increase −1 FS frequency on all reporters, albeit not always by the same degree. In contrast, +1 FS frequency is decreased in the G28D strain, while it is not affected in other strains. The stop codon readthrough is affected in a more varied way.

### uS5 mutations affect the mutation rate in *P. putida*

**Fig 5:**
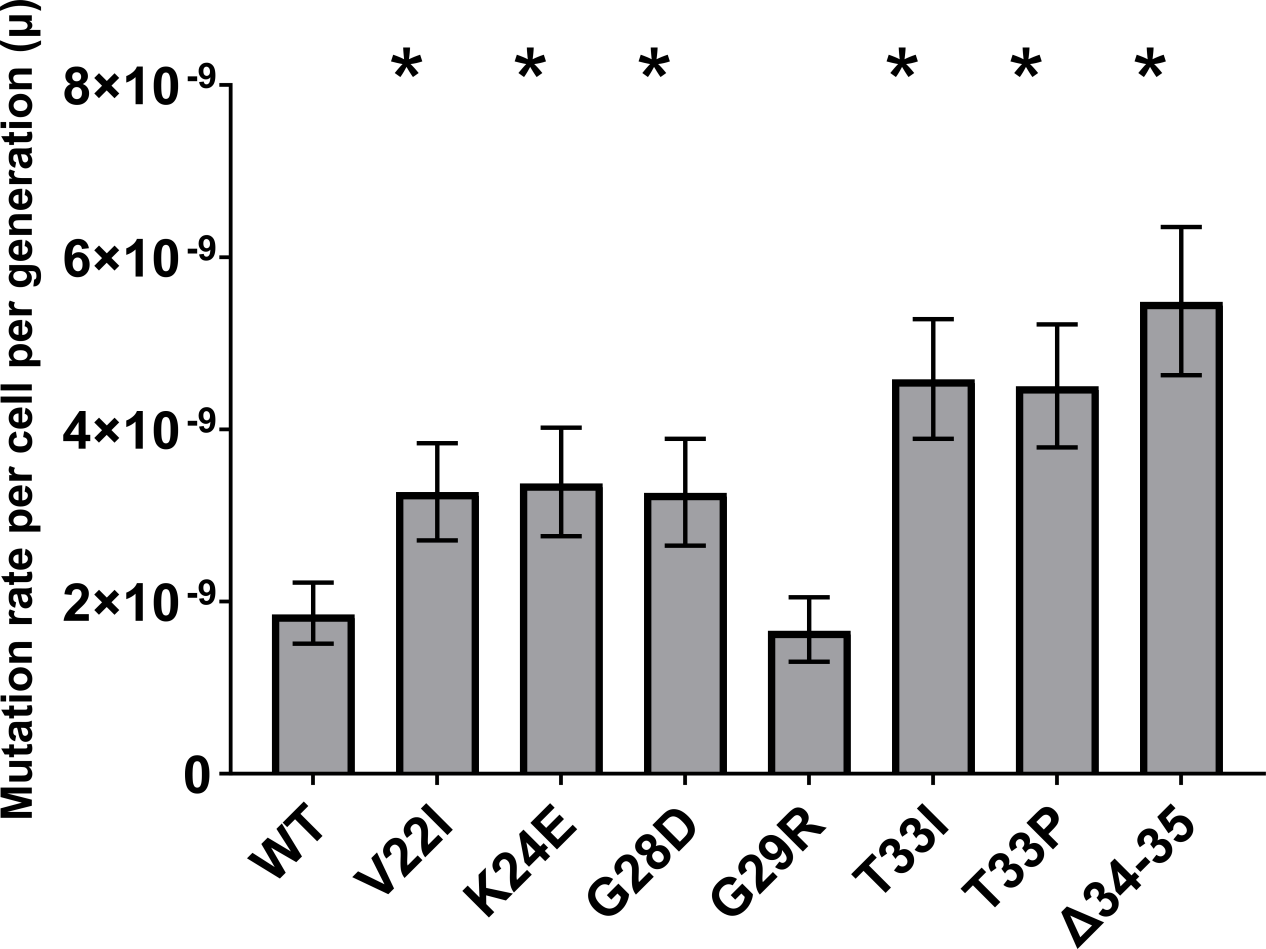
Comparison of mutation rates (μ) per cell per generation in WT *P. putida* PaW85 and uS5 mutant strains, based on rifampicin assay with 95% CI; ‘*’ indicates *P*-value < 0.001; *n* ≥ 28.

We estimated spontaneous mutation rates (μ, per cell per generation) from Rifᴿ fluctuation tests in WT PaW85 and the uS5 mutants. The results are shown in Fig. 5. All the investigated mutants of the uS5 protein had a significantly increased mutation rate, apart from G29R, which exhibited no change when compared to the wild type. The mutation rate increased threefold as a result of the deletion of residues 34–35 of uS5. Alterations V22I, K24E, and G28D displayed rather similar effects on mutation rate, an approximate twofold increase when compared to the wild type, and T33I and T33P strains had a 2.5-fold increase. All these effects were statistically significant.

It has been previously reported that mistranslation alone can be sufficient to achieve phenotypic rifampicin resistance without genetic mutations in the *rpoB* gene (28). To confirm whether rifampicin-resistant colonies were true mutants, the *rpoB* gene was sequenced in the case of 30 randomly selected Rif^R^ colonies of three strains with elevated mutation rate (K24E, T33P, and ∆34-35) that had emerged from independent cultures. The presence of a mutation in the *rpoB* gene was validated in all of the studied Rif^R^ mutants, confirming that they were true genetic mutants. The location of the mutation within the *rpoB* gene for all the mutants is shown in Table 1. There was a significant bias toward A → G transitions in all of the studied uS5 mutant strains. The A → G transitions are also prevailing in *P. putida* wild-type strain PaW85 (29). However, although the location of mutation hotspots within the *rpoB* gene remained similar to that of the wild type, the proportion of a specific A → G transition in *rpoB* position 1562 had increased significantly in the uS5 mutants. For the analyzed uS5 mutants, this mutation represented 60%–70% of all detected mutations, while the previous screen in the wild-type PaW85 had observed a relative frequency of 37.7%.

**TABLE 1:** Mutations in the *rpoB* gene in wild-type *P. putida* and uS5 mutants K24E, T33P, and del34-35^*a*^

Position in the *rpoB* gene	nt change	aa change	wt^*b*^	K24E	T33P	del 34–35
1547	T → C	L516P	0.0	0.0	3.3	0.0
1550	C → T	S517F	1.2	3.3	6.7	3.3
1553	A → G	Q518R	8.4	3.3	0.0	3.3
1553	A → T	Q518L	11.4	0.0	0.0	3.3
1561	G → A	D521N	0.6	3.3	3.3	0.0
1562	A → G	D521G	37.7	66.7	70.0	63.3
1562	A → T	D521V	0.0	0.0	0.0	3.3
1580	C → T	S527F	3.0	3.3	0.0	3.3
1591	C → T	H531Y	3.6	0.0	3.3	0.0
1592	A → T	H531L	3.6	0.0	0.0	3.3
1592	A → G	H531R	10.8	13.3	13.3	6.7
1607	C → T	S536F	9.6	3.3	0.0	10.0
1613	T → C	L538P	0.0	0.0	0.0	0.0
1706	C → T	P569L	0.0	3.3	0.0	0.0
Total number of sequenced mutants			167	30	30	30

^*a*^ Number shown is percentage from all detected rpoB mutations within the strain.

^*b*^ Gray shading indicates results from reference 29.

In summary, mutations in the uS5 protein significantly increased the mutation rate, with the most substantial effect observed in the ∆34-35 strain. The mutation spectrum in the *rpoB* gene remained consistent with our previous findings, but the A → G transition at position 1562 was more frequent in uS5 mutants, suggesting an altered mutation pattern.

### Effect of uS5 mutations on growth characteristics of *P. putida*

Growth parameters were determined for the wild-type *P. putida* strain and the strains carrying the uS5 mutant alleles when grown in a liquid glc + CAA medium. Calculated generation times and length of lag phase based on the growth curves are presented in Table 2 (representative graph of growth curves and graph with maximum growth rate are available in Fig. S1 and S2, respectively). We observed that uS5 alterations caused a slightly longer generation time when compared to the wild-type allele, with approximately 15%–20% increase. The effect was statistically significant in the uS5 mutant strains K24E, G28D, T33I, T33P, and ∆34-35. Differences in the length of the lag phase were statistically nonsignificant.

**TABLE 2:** Mean value of growth parameters with CI 95%^*a*^

Growth parameter	Value for strain							
	wt	V22I	K24E	G28D	G29R	T33I	T33P	∆34-35
Generation time (min)	31.94 ±1.92	36.42 ±1.50	37.88* ±1.84	38.20* ±2.02	35.78 ±1.94	37.52* ±1.76	38.92* ±2.28	36.63* ±1.65
Lag time (min)	32.96 ±9.19	35.93 ±8.51	23.56 ±6.46	31.49 ±8.42	44.68 ±9.69	31.22 ±9.01	38.7 ±9.28	39.06 ±8.84

^*a*^ * indicates *P*-value < 0.05 compared to the wild type.

**Fig 6:**
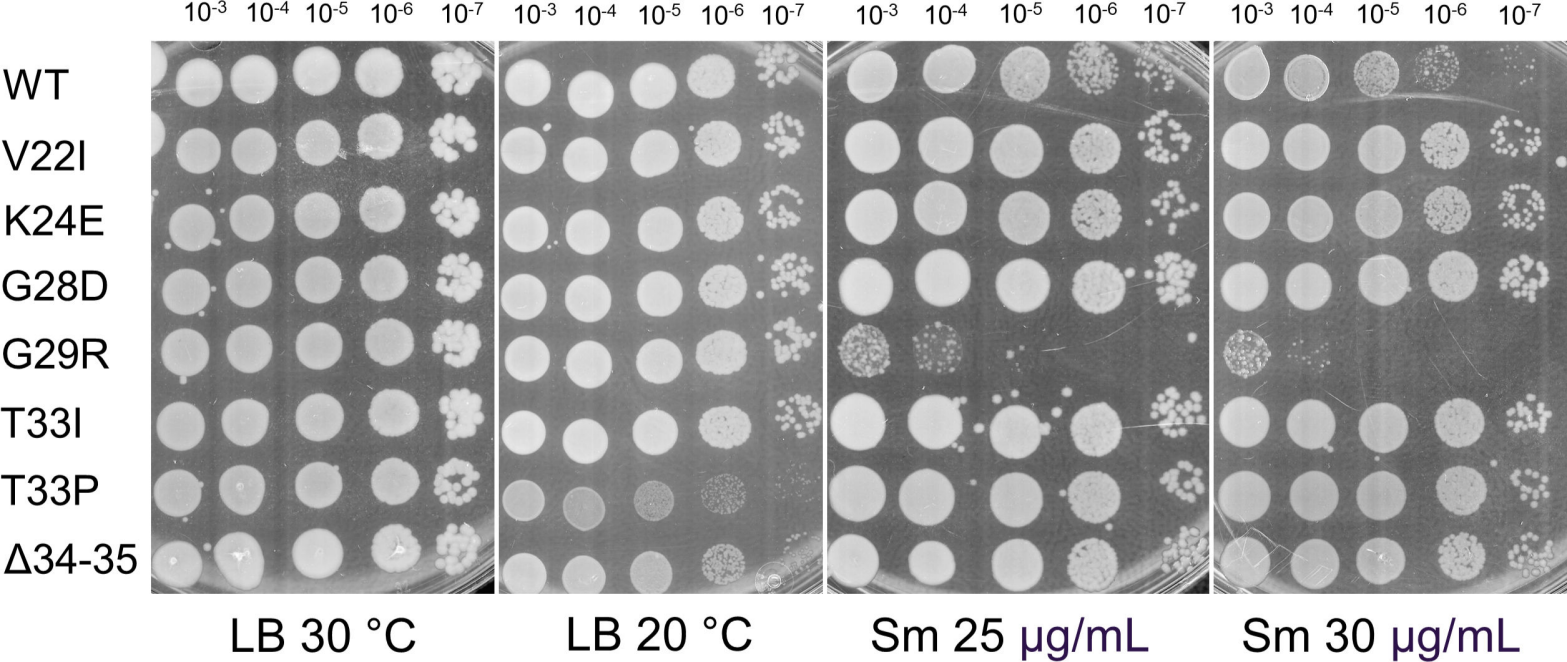
Dilution spot assay of the wild-type *P. putida* (strain PaW85) and the strains carrying uS5 mutant alleles. All images were captured after 24 hours of incubation.

The growth phenotypes were further assessed using the dilution spot assays on LB agar plates incubated at 30°C and 20°C, as well as at sublethal streptomycin concentrations (25 µg/mL and 30 µg/mL), given that selection used the 30S-targeting spectinomycin and streptomycin likewise targets the 30S subunit; none of the uS5 mutants were streptomycin resistant. Differences in colony formation between the wild-type strain and the strains carrying uS5 mutant alleles under these conditions are shown in Fig. 6.

Notable differences were observed between LB plates incubated at 30°C and 20°C for the strains T33P and ∆34-35. At 20°C, colonies were almost absent from the 10^−7^ dilution, while spots from lower dilutions appeared more transparent compared to the corresponding spots at 30°C of the same strains and the wild-type strain at 20°C, indicating reduced viability or slower growth at the lower temperature.

On plates containing sublethal concentrations of streptomycin, distinct viability patterns were observed. At 30 µg/mL streptomycin, all uS5 mutants—except G29R—exhibited slightly increased viability compared to the wild type, with larger and more numerous colonies. In contrast, the G29R mutant showed significantly reduced viability at both 25 µg/mL and 30 µg/mL, indicating increased sensitivity to streptomycin.

## DISCUSSION

In this study, a series of spectinomycin-resistant *P. putida* uS5 mutants were isolated and analyzed for translation accuracy and mutation rate. Investigated mutants, except for G29R, significantly increased the spontaneous mutation rate (Fig. 5). While the uS4-coding gene *rpsD* has been previously reported as a mutator gene (22), our data indicate that the uS5-coding gene *rpsE* also emerges as a novel mutator gene. This is consistent with observations in *E. coli*, which demonstrated that error-prone ribosomes provoke a mutator phenotype (22). uS4 mutant in *E. coli* activated a translational stress-induced mutagenesis pathway, elevating genome mutation rates (22). The exception, G29R, is revealing: G29R was quite error-prone in our translation assays yet produced no mutator effect, suggesting that not all errors equally trigger mutagenesis, which has also been shown in *E. coli* (30). Zheng et al. showed that mistranslation can enhance the deleterious effect of DNA mutations and, doing so, can lead to these cells being strongly competed out of the population, thus seemingly reducing the fixation of mutations (31).

Our data establish that *P. putida* strains with mutant uS5 protein exhibit a similar mistranslation phenotype reported in *E. coli* (20) and *S. typhimurium* (32) uS5 mutants. While most ram mutations cluster at the uS4-uS5 interface (near the C-terminal end for the uS5 protein), our data align with an *E. coli* study describing loop 2 alteration affecting decoding (23). Specifically, the G28D mutant in *E. coli* was shown to exhibit increased +1 and −1 frameshifting and stop codon readthrough (23). In our studies in *P. putida*, a mutant of the same residue (G29R) also exhibited increased stop codon readthrough and +1 and −1 frameshifting, albeit to a smaller degree (Fig. 3 and 4). Interestingly, the fidelity profiles of individual mutants varied substantially, with some (e.g., Δ34–35 and T33I/P) showing divergent effects on stop-codon readthrough and frameshifting. Loop 2 likely helps orient mRNA at the A-site for accurate frame maintenance (33). Experimental evidence from *E. coli* confirms that single-residue substitution (such as F30V) in uS5 protein can confer error-restrictive (hyperaccurate) ribosomal phenotype (20). This supports the view that loop 2 acts as a tunable element within decoding machinery: structural perturbations can allow for more frameshifting while causing a stricter control of stop codons. Overall, our data suggest that the examined uS5 mutations in the loop 2 region predominantly influence −1 frameshifting and stop codon readthrough, with comparatively minor effects on +1 frameshifting. Compared with tRNA-modifying enzymes (e.g., TruA/RluA), which we have previously reported and produce more restricted, context-dependent effects on translational fidelity (17), uS5 alterations act directly on the ribosome and yield broader shifts in decoding.

Translation errors could also increase mutation frequency in subpopulations of cells by compromising a correction of DNA replication errors, due to producing malfunctioning DNA proofreading and/or DNA repair enzymes, and thus accelerate population adaptability in fluctuating environments (34, 35). The malfunction or deficiency of DNA repair pathway enzymes or the activity of DNA polymerases has been shown to affect the molecular spectrum of mutations (8, 36–39). We have recently reported that the lack of pseudouridylation activity of TruA or RluA elevated mutation frequency in *P. putida* by a mechanism that was not clearly associated with error-prone DNA synthesis or malfunctioning of DNA repair functions (18). Although we did not observe any significant changes in the spectrum of Rif^R^ mutants between the *P. putida* wild-type strain and its TruA- or RluA-deficient derivatives (18), analysis of the spectrum of Rif^R^ mutants in the *rpsE* mutant strains of *P. putida* in the current study revealed a clear mutational hotspot A → G at position 1562 in the *P. putida rpoB* gene (Table 1). The genome of *P. putida* encodes, in addition to the replicative DNA polymerases Pol I and Pol III, a set of specialized DNA polymerases, Pol II, Pol IV, and DnaE2 (40–42). We have previously shown that the action of specialized DNA polymerases in *P. putida* can affect the spectrum of mutations (39). It has been demonstrated that in *E. coli,* mistranslation activates the SOS response (30, 43). Notably, in the *P. putida* strain lacking DNA Pol I, the frequency of A-to-G transitions at position 1562 increased more than 10-fold upon UV-irradiation, which induces the SOS response, whereas the frequency of these particular transitions decreased about 9-fold in Pol I-deficient cells when DnaE2 was absent (39). Different from the other specialized DNA polymerases in *P. putida*, the expression of DnaE2 is highly inducible by DNA damage (40). Thus, one may speculate that translational errors could affect the activity of the full set of DNA polymerases in a cell and thereby the frequency of replication errors at specific sites.

The regions of the nucleotide sequences of the *rpoB* gene containing Rif^R^ mutations are highly conserved between *E. coli* and *Pseudomonas* species (29). The A nucleotide at position 1562 in the *P. putida rpoB* gene corresponds to A at position 1547 in *E. coli rpoB*. As mentioned above, the proportion of the A → G transitions at position 1562 was increased in the *rpsE* mutant *P. putida* strains in comparison with the *P. putida* wild-type strain. The experiments in *E. coli* suggested that NDP kinase (Ndk) deficiency stimulates polymerase errors that lead to the A → G transitions at position 1547 in *E. coli rpoB*, and these transitions became even more evident when the DNA mismatch repair (MMR) was also disabled (44). Ndk is a housekeeping enzyme that balances cellular nucleoside triphosphate (NTP) pools by catalyzing the reversible transfer of γ-phosphate from NTPs to NDPs (45, 46). Lu et al. (47) found that the *ndk* deletion mutant contains steady-state pools of dCTP up to 23-fold higher than those in the wild-type strain, and the dGTP pool was also expanded sevenfold. dNTP pool imbalances can stimulate mutagenesis either as a result of insertion errors, as a dNTP present in excess can stimulate non-Watson base pairing, or affect DNA replication proofreading activity, as a dNTP in excess can extend from a mismatched 3′ terminal nucleotide its removal (45, 48). Hence, the existing direction for future work is to study whether translation errors could increase mutation frequency by impairing the Ndk kinase activities.

Spectinomycin selection has been previously used to isolate uS5 mutants in *E. coli* (25) and was successfully used in the current study to obtain uS5 mutants in *P. putida*. Spectinomycin binds to the ribosome near the interaction site of helix 34 of 16s rRNA and loop 2 of uS5 protein and inhibits the translocation of tRNA from the A site to the P site (49, 50). Whereas 60 µg/mL spectinomycin suffices for selection in *E. coli* (25) (and matches MICs in *N. gonorrhoeae* [51]), *P. putida* required 1,750 µg/mL. This higher threshold is unlikely to reflect altered targets of the drug: the nucleotide composition of helix 34 (positions 1046 to 1067 and 1189 to 1211, *E. coli* numbering) is identical in *P. putida*, and residues in and around uS5 loop 2 (residues 20–31 based on *E. coli* numbering) are well conserved. Rather, it likely reflects *Pseudomonas* spp. intrinsic higher tolerance to antibiotics, mainly owing to the lower permeability of the outer membrane (52) and a larger number of efflux pumps (53).

The screen yielded seven uS5 loop-2 variants with amino acid substitutions or deletions in the positions 22–35 of the uS5 protein. These positions are akin to what has been reported for spectinomycin-resistant uS5 mutants in *E. coli* (25, 54)*, N. gonorrhoeae* (51), *S. suis* (55), and *P. multocida* (56). Conversely, an *E. coli* strain with an altered amino acid (F30V) in the same region of uS5 protein had increased susceptibility to spectinomycin compared to wild type, as was the case for many strains with alterations further toward the C-terminus (20). Our results indicate that a variety of alterations in and around the loop 2 of uS5 confer resistance to spectinomycin, probably due to perturbation of interactions of the drug with its target site. Because streptomycin targets the 30S decoding center near uS12 (distinct from the spectinomycin site), we tested sublethal streptomycin (25 and 30 µg/mL) to probe functional consequences. We observed slightly increased tolerance to the drug in uS5 mutants K24E, G28D, T33I, T33P, and ∆34-35 when compared to wild type, whereas G29R was hypersensitive (Fig. 6). While not in close vicinity to uS12, these residues may destabilize uS5 and, in doing so, affect the somewhat distant uS4-uS5 interface, which is critical for switching between “open” and “closed” ribosomal state and thus may indirectly modulate the susceptibility of uS12 to streptomycin. The bidirectional effect between closely positioned residues hints at the complexity of the allosteric effect of uS5 mutants on streptomycin binding.

The reduced viability at 20°C of uS5 mutants T33P and ∆34-35 (Fig. 6) parallels the cold-sensitive phenotype of the *E. coli* uS5 mutant (G28D), which was attributed to an assembly defect of 70S ribosomes (23). However, interestingly, the alteration of the same residue in *P. putida* in the G29R strain (G28 in *E. coli* numbering is G29) evoked no cold sensitivity, as was the case for all the other investigated *P. putida* strains, among which was another strain, where threonine in the 33rd position had been replaced by isoleucine. At 30°C, in addition to T33P and ∆34-35, a generation time increase of about 10%–20% was observed for strains K24E, G28D, and T33I, further illustrating the fitness cost of mutations in the *rpsE* gene (Table 2). This is potentially due to ribosomal assembly delay, caused by impaired uS5-16S rRNA interactions and improper maturation of 16S rRNA (21). In *E. coli,* some uS5 mutants have displayed significantly increased doubling time (up to 100%) while a number of Ram-phenotype uS5 mutants without increased growth time have also been isolated (20, 23).

The loss of fitness is widely associated with increased mistranslation under optimal growth conditions, but errors in translation can also be an important component of individual variation with significant consequences for stress survival (1, 2, 30, 57). For example, mistranslation-mediated phenotypic resistance to rifampicin in *Mycobacterium tuberculosis* was largely, although not exclusively, due to protein variants that resulted in drug resistance (28); mistranslation induced by a mutation in the ribosomal protein uS4 triggers a RpoS-mediated oxidative stress response in *E. coli* (58). Taken together, these findings underscore the dual nature of translational errors as both a liability and an adaptive mechanism. Our study adds to this understanding by demonstrating how specific mutations in ribosomal protein uS5 modulate mistranslation and influence bacterial fitness and mutagenesis, highlighting the complex crosstalk between translational fidelity and adaptability in fluctuating environments.

## MATERIALS AND METHODS

### Bacterial strains and plasmids

The bacterial strains and plasmids used in this study are listed in Table 3.

**TABLE 3:** Bacterial strains and plasmids used in this study

Strain or plasmid	Description	Source
*P. putida* strains		
PaW85	Wild type, isogenic to *Pseudomonas putida* strain KT2440	(59, 60)
V22I	PaW85 uS5 (*rpsE, PP_0471*) mutant with V22I substitution	This study
K24E	PaW85 uS5 (*rpsE, PP_0471*) mutant with K24E substitution	This study
G28D	PaW85 uS5 (*rpsE, PP_0471*) mutant with G28D substitution	This study
G29R	PaW85 uS5 (*rpsE, PP_0471*) mutant with G29R substitution	This study
T33I	PaW85 uS5 (*rpsE, PP_0471*) mutant with T33I substitution	This study
T33P	PaW85 uS5 (*rpsE, PP_0471*) mutant with T33P substitution	This study
∆34-35	PaW85 uS5 (*rpsE, PP_0471*) mutant with deletion of residues F34-T35	This study
Plasmids		
pSEVA AD2	Reporter plasmid with a −1 frameshift signal between the Rluc and Fluc gene, Km^R^, Gm^R^	(17)
pSEVA AD7	Reporter plasmid with a −1 frameshift signal between the Rluc and Fluc gene, Km^R^, Gm^R^	(17)
pSEVA 304 UAG	Reporter plasmid with a premature stop codon (UAG) in the Fluc gene, Km^R^, Gm^R^	(17)
pSEVA 417 UGA	Reporter plasmid with a premature stop codon (UGA) in the Fluc gene, Km^R^, Gm^R^	(17)
pSEVA UUC-	Reporter plasmid with a slippery sequence and a −1 frameshift signal between the Rluc and Fluc gene, Km^R^, Gm^R^	(17)
pSEVA UUC+	Reporter plasmid with a slippery sequence and a +1 frameshift signal between the Rluc and Fluc gene, Km^R^, Gm^R^	(17)

### Selection of spectinomycin-resistant strains

A portion (100 µL) of the overnight culture of *P. putida* PaW85 was plated on LB agar plates containing spectinomycin (1,750 µg/mL) and incubated for 48 hours at 30°C to select for spontaneously arisen *rpsE* mutants. Mutations were confirmed by WGS.

### Dual-luciferase translation assay

Cells carrying reporter system (Fig. 2) plasmids were grown overnight at 30°C in 1.5 mL glc + CAA medium with kanamycin (50 µg/mL). Cultures were diluted to an OD_600_ ~0.1 into 2 mL of fresh medium with kanamycin and 0.5 mM IPTG. After 3 hours at 30°C, cells were pelleted, flash-frozen, and resuspended in 400 µL Passive Lysis buffer (Promega).

For measurement, 50 µL of extract was assayed for Fluc activity, followed by a 50-fold dilution and Rluc assay. Luminescence was measured using a TECAN Infinite Pro M200 plate reader (Fluc: 10 s read after 2 min, Rluc: 10 s read after 4 min). Reporter outputs were normalized by dividing each mutant’s Fluc/Rluc ratio by the corresponding (same reporter) Fluc/Rluc ratio of the wild-type strain.

### Rifᴿ fluctuation assay and mutation rate estimation

Spontaneous mutation rates (μ, per cell per generation) were estimated using fluctuation data from the Rif^R^ assay. Cultures were grown about 6 hours into late log phase in glc + CAA medium, diluted 10^5^ times, and distributed into 10 tubes (2.3 mL each). After 20–22 h of growth, ~5 × 10⁸ cells from each culture were plated onto LB agar containing 100 µg/mL rifampicin. CFU counts were obtained from parallel plates without rifampicin. Rifᴿ colonies were counted after 48 h at 30°C. Mutation rates were calculated using the MLE MUtation Rate calculator (mlemur) (61).

### Growth analysis and stress tolerance assay

For growth analysis, cells were grown overnight in glucose + CAA medium, then diluted to an OD_600_ of 0.1 before transferring 100 µL of the diluted culture to the 96-well plate. Optical density data were collected over 16 hours at 10-minute intervals. Data were analyzed using QurvE software with built-in tools to obtain growth parameters (62). Stress tolerance was assessed using dilution spot assays, with 5 µL of overnight cultures spotted onto LB agar plates. Plates were incubated at 30°C and 20°C, as well as on LB plates supplemented with streptomycin (25 µg/mL and 30 µg/mL).

### Sequencing and mutation confirmation

For WGS, genomic DNA from uS5 mutants was extracted using the Thermo Scientific GeneJET Genomic DNA Purification Kit. Sequencing was performed on the Illumina MiSeq platform with 100× coverage. Sequencing reads were cleaned and filtered with fastp (63). Mutations were detected with breseq v.0.35.0 (64) using *P. putida* KT2440 (NC_002947) as a reference. For the Rif^R^ mutation spectrum, random rifampicin-resistant mutants (one per plate) were selected and sequenced with primer PPrpoB1 (sequence: GGCGGAAAGCGAAGGCCTG) to verify the mutation in the *rpoB* gene.

### Statistical analysis

The Shapiro-Wilk test was used to determine the normality of the data set. As the data did not follow a normal distribution, non-parametric methods were used to compare different data sets. Kruskal-Wallis test was performed, followed by Dunn’s post-hoc test. Calculations were performed using GraphPad Prism 10.

## Data Availability

Raw whole-genome sequencing reads have been deposited in the NCBI Sequence Read Archive (SRA) under BioProject accession PRJNA1337407. The data that support the findings of this study are available from the corresponding author upon reasonable request.

## SUPPLEMENTAL MATERIAL

Supplemental figures jb.00334-25-s0001.docx Figures S1 and S2.

ASM does not own the copyrights to Supplemental Material that may be linked to, or accessed through, an article. The authors have granted ASM a non-exclusive, world-wide license to publish the Supplemental Material files. Please contact the corresponding author directly for reuse.
