# Supplementary material for: Mutations in ribosomal protein uS5 alter translation fidelity and mutagenesis in Pseudomonas putida
Source: J Bacteriol. 2025 Nov 12;207(12):e00334-25. doi: 10.1128/jb.00334-25 (PMC12713390; doi:10.1128/jb.00334-25)
Supplement: Supplemental figures — Figures S1 and S2. [file jb.00334-25-s0001.docx]

**Supplementary figures and tables**

**Figure S 1**. Bacterial growth based on optical density of the culture (λ=600 nm) over 9 hours. Colors depict different strains, the dark line is the mean value and lighter shade around it depicts the 95% CI range, n ≥ 23.

******

**Figure S 2**. Maximum growth rate of the wild-type P. putida (strain PaW85) and the strains with mutant uS5 proteins with CI 95%, ‘*’ indicates p-value <0.05, n ≥ 23.
